# Epidemiology of dengue fever in Hanoi from 2002 to 2010 and its meteorological determinants

**DOI:** 10.3402/gha.v7.23074

**Published:** 2014-12-08

**Authors:** Dao Thi Minh An, Joacim Rocklöv

**Affiliations:** 1 Department of Epidemiology, Institute for Preventive Medicine and Public Health, Hanoi Medical University, Hanoi, Vietnam; 2 Epidemiology and Global Health, Department of Public Health and Clinical Medicine, Umeå University, Umeå, Sweden

**Keywords:** dengue fever, epidemiology, time-series analysis, weather, climate

## Abstract

**Background:**

Dengue fever (DF) is a growing public health problem in Vietnam. The disease burden in Vietnam has been increasing for decades. In Hanoi, in contrast to many other regions, extrinsic drivers such as weather have not been proved to be predictive of disease frequency, which limits the usefulness of such factors in an early warning system.

**Aims:**

The purpose of this research was to review the epidemiology of DF transmission and investigate the role of weather factors contributing to occurrence of DF cases.

**Methods:**

Monthly data from Hanoi (2002–2010) were used to test the proposed model. Descriptive time-series analysis was conducted. Stepwise multivariate linear regression analysis assuming a negative binomial distribution was established through several models. The predictors used were lags of 1–3 months previous observations of mean rainfall, mean temperature, DF cases, and their interactions.

**Results:**

Descriptive analysis showed that DF occurred annually and seasonally with an increasing time trend in Hanoi. The annual low occurred from December to March followed by a gradual increase from April to July with a peak in September, October. The amplitude of the annual peak varied between years. Statistically significant relationships were estimated at lag 1–3 with rainfall, autocorrelation, and their interaction while temperature was estimated as influential at lag 3 only. For these relationships, the final model determined a correlation of 92% between predicted number of dengue cases and the observed dengue disease frequencies.

**Conclusions:**

Although the model performance was good, the findings suggest that other forces related to urbanization, density of population, globalization with increasing transport of people and goods, herd immunity, government vector control capacity, and changes in serotypes are also likely influencing the transmission of DF. Additional research taking into account all of these factors besides climatic factors is needed to help developing and developed countries find the right intervention for controlling DF epidemics, and to set up early warning systems with high sensitivity and specificity. Immediate action to control DF outbreak in Hanoi should include an information, communication, and education program that focuses on training Hanoi residents to more efficiently eliminate stagnant puddles and water containers after each rainfall to limit the vector population growth.

The incidence of dengue fever (DF) has grown dramatically around the world in recent decades. Over 2.5 billion people – over 40% of the world's population – are now at risk. WHO currently estimates there may be 50–100 million dengue infections worldwide every year (1). The Intergovernmental Panel of Climate Change (IPCC) warned that up until 2080, there may be 1.5–3.5 billion people worldwide who have to face the risk of DF infection due to climate change and the effects of the earth warming (2). New estimates show this may be substantially underestimated if economic development was less positive (3, 4). DF appeared in Vietnam in the late 1950s. Since then, DF became endemic with seasonal peaks occurring yearly and with a repeating epidemic pattern ranging from 4 to 10 years (peaks in 1983, 1987, and 1998). The milestone of DF epidemics in Vietnam was the large-scale outbreak in 1998 that impacted 57 out of total 61 provinces with the number of infected patients reaching 234,920 including 377 deaths. In response to this crisis, the Vietnam Government has approved the national dengue prevention program with the regions. The northern dengue control program, with its head office located in the National Institute of Hygiene and Epidemiology (NIHE), was established and started in 1999 (March/1999) (5). Since then, Vietnam appears to have controlled DF outbreaks for a long period; however, in 2009, the country once again experienced a DF outbreak in which DF cases peaked at 74,000 cases in October 2009 (increased by 17% compared with the same period in 2008) including 58 reported deaths (6).

Hanoi, one of the two biggest cities in Vietnam, experienced 16,263 DF cases in 2009 that spread to all of Hanoi's districts and occupied 87% total DF cases in the northern area. The number of DF cases was 6.7 times compared with the number in 2008 in Hanoi. The Ministry of Health noticed that the outbreak in Hanoi in 2009 was the most severe outbreak during the past 10 years with 121 cases per 100,000 population (7).

According to the Ministry of Health's statistics, the years that Vietnam experienced huge DF outbreaks during the past 10 years (1987, 1998, 2009) coincided with years of increased El Nino and La Nina activity (8). Recently, studies have been published on DF hot spots and the disease dynamics dispersion of DF over the period 2004–2009 in Hanoi, Vietnam, and one study quantifying the Emergence of Dengue in Hanoi, Vietnam: 1998–2009 (9, 10). However, studies to understand how much weather factors influenced the DF epidemic, especially in Hanoi, are scarce. Such studies are important to provide useful evidence for DF control programs through the development of early warnings (11). Therefore, the purpose of this study was to investigate characteristics of DF cases in Hanoi in relation to variation of weather factors over the period 2002**–**2010.

## Materials and methods

### Study area

Hanoi is the capital of Vietnam, ranked second among the country's most populous cities. It has been the most important cultural and political center with a population estimated at around 3 million spread over nine inner and five outer districts in 2008. On 1 August 2008, one further inner and 14 outer districts merged with the metropolitan area of Hanoi which increased Hanoi's total area to 334,470 hectares in 29 subdivisions with the new population reaching 6,232,940, effectively tripling in size. Hanoi's transportation density of people and goods remained second of the nation.

In 2008, Hanoi experienced heavy rain and floods (12). In general, Hanoi is characterized by a warm humid subtropical climate with plentiful precipitation peaking in the summer season and averaging 114 rainfall days per year in modern times. The city experiences a typical climate of northern Vietnam with two separated seasons: hot and humid summers, and relative to other parts of the nation cold and dry winters. Summers, lasting from May to September, are hot and humid with an average temperature of 28.1°C, receiving the majority of the annual rainfall. The winters, lasting from November to March, are relatively mild, dry (in the first half) or humid (in the second half) with an average temperature of 18.6°C. Hanoi also has two transition periods in April and October, that is, spring and fall. The temperature variation width ranges from 8 to 37°C (13).

### Data collection

DF was categorized in group B of infectious diseases in which the Infectious Diseases Act in Vietnam stipulates it mandatory that DF must be notified within 24 hours of diagnosis by all medical clinics and laboratories (14). Circulars of guidance on notification, communication, and reports of infectious diseases regulates that in each province/city, DF cases must be reported *weekly* by the commune health centers and the district hospitals to district health centers which reports to the department of preventive medicine at provincial level (PDPM) using WHO 2009 criteria of DF definition (Annex 1). The weekly reported DF cases were then collapsed into monthly aggregated numbers by PDPM and reported to the NIHE (15). We extracted monthly aggregated DF data reported by 14 old districts of Hanoi to the Hanoi PDPM from 2002 to 2010. We also obtained daily temperatures in centigrade, relative humidity in percentages and rainfall in millimeters for 2002–2010, reported by Lang center (the Hanoi Centre of Hydrometeorology before 2008). Weather data were collapsed into monthly mean values. The monthly aggregated DF data were merged with the monthly mean weather data for the epidemiological time-series analysis.

### Statistical methods

A dengue outbreak is characterized by the occurrence of excess DF cases compared to what would normally be expected in a defined community, geographical area or season. Characteristics of DF epidemic from 2002 to 2010 were described and tested by including variables for the estimation of time trends. Variability of temperature, rainfall, humidity and DF cases were hypothesized to precede the upsurge or decay of DF cases with a lag of up to 6 months. Population changes were adjusted for in the denominator of the dengue count series (offset). Spearman correlation was estimated between DF cases and each five weather factors to identify the most influential preceding months (lag times) that influenced the occurrence of DF cases. The lag variables with correlations running from ∣0.3∣ to ∣1∣ were selected to be independent variables in a subsequent negative binomial regression model. Bonferroni corrections per number of lag tests were conducted to adjust for multiple testing with an adjusted significance level at 0.05/6 (16). The negative binomial regression model was chosen to relax the assumption of mean and variance equality in the Poisson distribution of counts data. In 2009, Yang indicated that mortality rates of adult mosquitoes increase with increasing temperature above 30°C (17). Fouque and Dibo indicated that heavy rainfall can potentially flush away larvae or pupae or the immature stage of Aedes mosquitoes. Heavy rainfall can also increase the mortality rate of adult mosquitoes (18, 19). Therefore, a threshold of 30°C and 450 mm rainfall was used for running piecewise linear spline functions with the hypothesis that there was a positive linear relationship between DF cases when temperature increases from 15 to 30°C and rainfall increases from 0 to 450 mm. Beyond 30°C and 450 mm, these relationships would be in reverse order (negative). Lag variables that were statistically significant in a simple negative binomial regression model would then be included in a multiple regression model. A manual stepwise model selection approach with forward inclusion was used to identify the most appropriate model based on the Generalized Cross Validation (GCV) scores and Akaike Information Criterion (AIC). A time variable was also used as an independent variable to control trends of DF cases over time not explained by the other variables. Predictions from the established models and its relationship to the observed dengue cases were evaluated based on Root Mean Square Error (RMSE), and correlation. We also validated the fit of models by performing residual diagnoses, and graphic examination.

The fitted models can be expressed as follows:

a $$\log\left(DF_{t}\right)=D_{0}+D_{temp}+D_{rain}+D_{AR}+D_{trend}+offset\left(\text{log}(pop)\right)$$

*Where t* refers to the month of the observation; (*DF*_*t*_) denotes the observed monthly DF cases during month *t*.

Thus,

D_*temp*_=lags of monthly mean temperature

D_*rain*_=lags of monthly mean rainfall

D_*AR*_=lags of auto-regression (DF cases)

D_*trend*_=a function of time trend (year)

offset(log(pop))=the DF denominator adjustment of mid-year population.

In addition, penalized cubic spline functions were fit to further explore non-linear patterns in additional models taking the form:

$$\log\left(DF_{t}\right)=D_{0}+s\left(D_{temp},df\right)+s\left(D_{rain},df\right)+s\left(D_{humid},df\right)+s\left(D_{AR},df\right)+s\left(D_{trend},df\right)+s\left(offset(\log(pop)),df\right)$$

Where *s(.)* denotes a smooth function; *df* represent degrees of freedom that are penalized in the model fitting from a start value of 10; *D*_*temp*_ *, D*_*rain*_ *, D*_*humid*_ *, D*_*AR*_ are the mean monthly temperature, rainfall, humidity, and DF case, respectively. *D*_*trend*_ represents factors for year of study period, respectively.

For all statistical tests, two-tailed tests were considered statistically significant with a p-value less than 0.05. All data manipulation were done in STATA and statistical analyses were performed in STATA and using the R package ‘mgcv’ (The R Foundation for Statistical Computing, version 3.0.0).

## Results

**Fig. 1:**
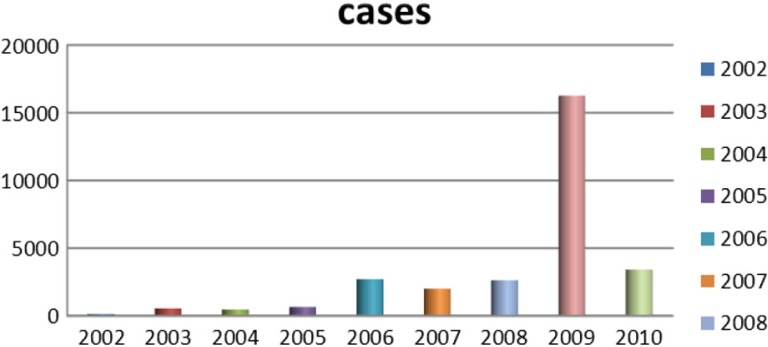
Annual distributions of DF cases in Hanoi (study period: 2002–2010).

**Map 1:**
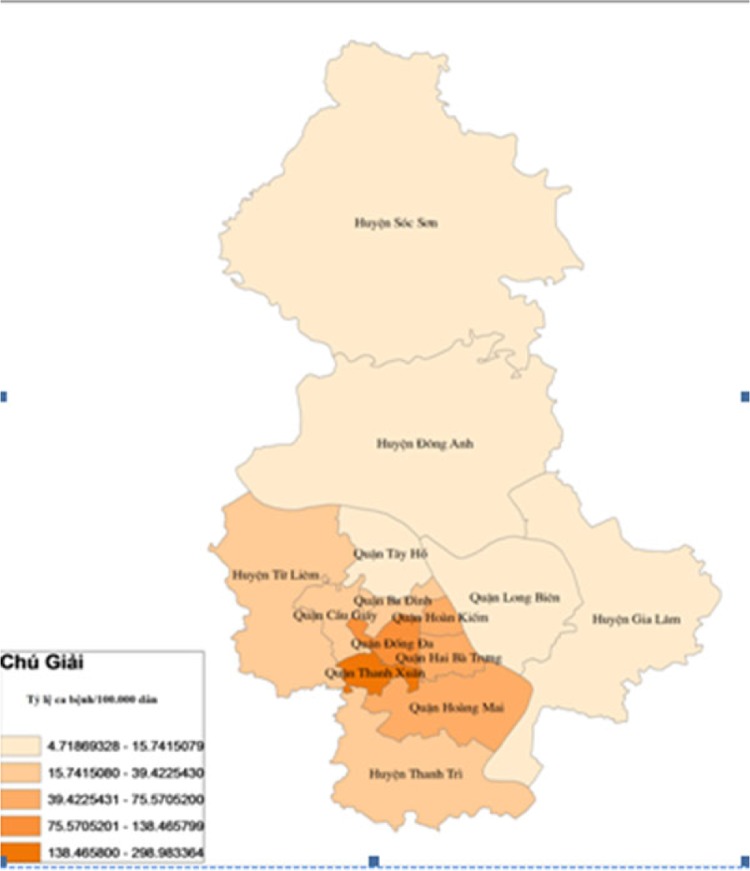
Distribution of DF cases in 14 districts of Hanoi from 2002 to 2010.

During the study period from January 2002 to December 2010, there were 28,793 DF cases in which more than 75% of them were aged between 15 and 44 years. Male cases were higher at all years. DF cases occurred mostly in inner districts (72.07%) and the rest belonged to outer districts. Within inner districts, four bordering districts faced recurrent outbreaks over the 9 years. These were Dong Da, Thanh Xuan, Hoang Mai, Thanh Tri, Hai Ba Trung. Within the outer districts, the two bordering areas Thanh Tri and Tu Liem suffered the highest number of DF cases (Map 1). DF cases increased from 125 cases in 2002 to 649 cases in 2005, and after that, DF cases increased with greater magnitude and intensity with the, at the time, record of 2,707 cases in 2006 to become even worse in 2009 with 16,268 cases. The rate of DF cases per 100,000 population per year increased significantly from 2002 to 2010 (p-value of trend test is 0.03) and numbers of DF cases per month increased significantly over 108 months of 9 years (*p*-value of trend test is <0.000). The highest dengue cases in the study period were reported in September and October 2009 with 4,145 and 4,120 cases, respectively. DF outbreaks occurred in Hanoi from 2006 to 2010 with the number of cases being 4.3, 3.3, 4.1, 25.6, and 5.4 times higher, respectively, compared with previous years (Fig. 1).

**Fig. 2:**
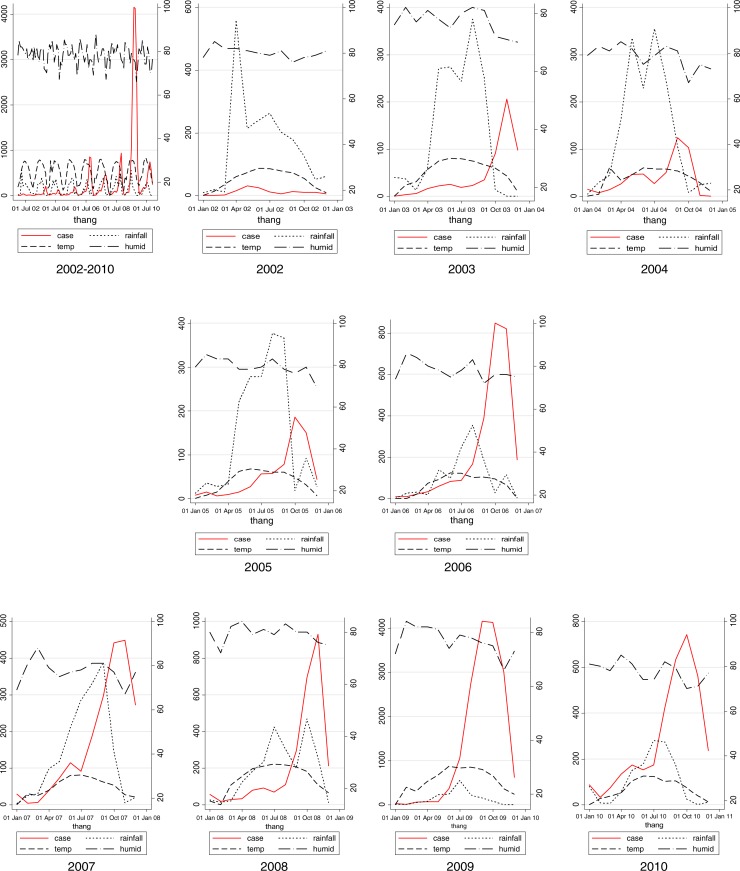
Whole period and monthly mean distribution of DF cases, rainfall, temperature, and humidity, Hanoi, 2002–2010.

This study indicated that in the period 2002–2010, DF cases generally occurred annually in a seasonal way, with the exception of 2002. The general pattern revealed a few DF cases that appeared sporadically from December of the previous year to March of the next year, increased gradually from April to July, peaked in September, October, and decreased quickly in November and December. A new circle of DF cases would occur the year after. It is easily observed graphically that before each peak of DF cases, there is always a preceding 1–2 months excessive rainfall (Fig. 2). As can be seen in Fig. 2, rainfall peaks in July and August, and then DF cases peak a few months later in September and October. In the period when rainfall was peaking, temperature were always around 20–30°C and humidity was around 70–80% (Fig. 2). Table 1 reveals that three weather factors are significantly correlated with DF cases on the basis of Spearman correlations. These are temperature, rainfall, and humidity. Temperature is significantly correlated with DF cases through lag 0–3, with the biggest correlation at lag 2 (*r*=0.53). Similarly, rainfall is significantly correlated with DF cases through lag 1–3 with the largest correlation at lag 2 (*r*=0.47). Humidity had only moderate correlation with DF cases at lag 0, while non-significant over the other lags times. Past DF cases were correlated with DF cases at the moment through lag 1–3, but with the highest correlation with *r*=0.84 at lag 1. There is a significantly strong positive correlations between DF cases and population with *r*=0.63. Quasi Poisson Regression showed that there is a significant and positive linear association between temperature and DF cases when temperature was below (≤) 30°C, but this association is reversed when temperature increased beyond 30°C. In contrast, there is only significantly positive linear regression between DF cases and rainfall when temperature was below (≤) 450 mm. Noticeably, only DF cases at lag 1 significantly precede risk of DF cases while temperature and rainfall preceded risks of DF cases by lag 1–3 (Table 1).

**Table 1:** Correlation and regression coefficients (negative binomial) between DF case and independent variables (bivariate Spearman rank correlations), Hanoi, 2002–2010

	Temperature			Rainfall			Case		Population		Humidity	
	
Lag time	Spearman's rho (p)	Spline (^o^C)	Quasi Poisson's coefficient (p)	Spearman's rho (p)	Spline (mm)	Quasi Poisson's coefficient (p)	Spearman's rho (p)	Quasi Poisson's coefficient(p)	Spearman's rho (p)	Quasi Poisson's coefficient (p)	Spearman's rho (p)	Quasi Poisson's coefficient (p)
Lag 0	0.28 (0)*	≤30	0.24 (0)*	0.14 (0.16)	na	na	na	na	0.63 (0)*		−0.35 (0)*	−.1209 (0.000)*
		>30	−0.25 (0.004)*		na	na	na			0.0007 (0)*		
Lag 1	0.47 (0)*	≤30	0.23 (0)*	0.36 (0)*	≤450	0.004 (0)*		0.002 (0.000)*	na			na
		>30	−1.94 (0.008)*		>450	−0.0008 (0.922)	0.84 (0)*			na	−0.10 (0.30)	
Lag 2	0.53 (0)*	≤30	0.22 (0)*	0.47 (0)*	≤450	0.006 (0)*		−0.001 (0.287)	na			na
		>30	−0.52 (0.447)		>450	−0.008 (0.21)	0.57 (0)*			na	0.077 (0.44)	
Lag 3	0.44 (0)*	≤30	0.15 (0)*	0.38 (0)*	≤450	0.006 (0)*						na
		>30	0.75 (0.362)		>450	−0.11 (0.104)	0.37 (0)*	0.00009 (0.884)	na	na	0.135 (0.17)	na
Lag 4	0.25 (0.01)*	na	na	na	na	na	0.27 (0.01)*		na	na	na	na
Lag 5	0.02 (0.81)	na	na	na	na	na	0.21 (0.03)*		na	na	na	na
Lag 6	−0.21 (0.03)	na	na	na	na	na	0.18 (0.07)		na	na	na	na

* s p≤0.05.

**Fig. 3:**
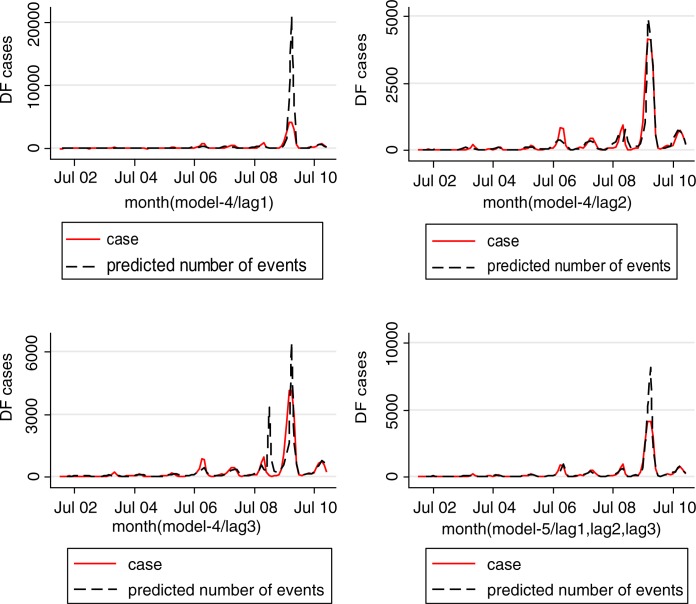
Predicted cases vs. observed DF cases in Hanoi, 2002–2010.

Four models were developed using the manual multiple forward stepwise regression analysis using lag 1–3 of predictors (Table 2). In the first step, we incorporated an annual time trend factor variable of the nine consecutive years (2002–2010), and developed model-1 considering rainfall and DF cases with the hypothesis that rainfall (lag 1–3) is a necessary condition for mosquito reproduction. In the second steps we put rainfall and temperature (lag 1–3) together in model-2 with the hypothesis that temperature may also play an important role in reproduction and proliferation of disease. The third step involved putting rainfall, temperature, and lag cases together in model-3 with the hypothesis that DF cases would contribute by an inbuilt momentum in the disease growth process after the onset of the epidemic. We also ran model-4 by putting rainfall, temperature, autocorrelation, and interaction among these three factors together in a model with the hypothesis that there would be an interaction among rainfall, temperature and DF cases that could make outbreak more explosive. We found that by different lag time (lag 1–3), model-1 demonstrated a capacity to explain a correlation of maximum 78.1% comparing the predictions of the model to the total variations in the occurrence of DF case, while correlations of model-2, model-3, and model-4 explained a Spearman correlation of the predicted values to the observed of maximum 85.1, 87.8 and 88%, respectively. Finally, a full model (model-5) including interactions between weather factors of lag 1–3 together with main effects was fitted to study more complex associations. In this model, the explanatory capability further increased over the previous four models and could explain a correlation of the predicted to the observed dengue cases to a correlation of 92%. We explored another model with an even higher lag period (>3); however, these models failed to add to explanatory capacity, and rather showed sharp declines in model fit. Hence, model-5 was chosen as the final model. While generating the model, all of the relevant assumptions were checked for assuring best possible model to be selected. For model-5, the AIC value equals 1,110.89 which is the lowest and similarly optimal value compared with those of model-4s from lag 1 to 3 running from 1,157, 1,157, and 1,183, respectively (Table 2). Moreover, its GCV score was the lowest compared with those of model-4 through lag 1–3 (45.2 against 58.9, 115, 171, respectively; see Table 2 and 3) while its RMSE score was the second compared with those of model-4 through lag 1–3 (33.63 against 205.95, 7.85, 1.25, respectively; see Table 2 and 3). Besides, graphs of the actual case against predicted values of model-4 at three lags of time and model-5 showed that model-5 performed the fitness of distribution of observed DF cases against predicted values (Fig. 3), and penalized spline function graphs of these exposure–response relationships are presented in Annex 2 to Annex 5, respectively.

**Table 2:** Stepwise multivariate regression between DF cases and influent factors, Hanoi, 2002–2010

		Model-1				Model-2				Model-3				Model-4			
		
Lag		Coef	*p*	[95% Conf. interval]		Coef.	*p*	[95% Conf. interval]		Coef.	*p*	[95% Conf. interval]		Coef.	*p*	[95% Conf. Interval]	
	p and r correlation	*p*<0.001; *r*=72.6				*p*<0.001; *r*=81.9				*p*<0.001; *r*=87.8				*p*<0.001; *r*=88; AIC=1157; RMSE=205.95; GCV=58.9			
	rain1lag1	0.00	0.00	0.00	0.01	0.00	0.88	0.00	0.00	0.00	0.23	0.00	0.00	0.00	0.79	0.00	0.00
Lag1	temp1lag1					0.22	0.00	0.16	0.28	0.18	0.00	0.13	0.23	0.19	0.00	0.13	0.24
	lagcase1									0.00	0.00	0.00	0.00	0.00	0.00	0.00	0.00
	interact1													0.00	0.13	0.00	0.00
	p and r correlation	*p*<0.001; *r*=78.1				*p*<0.001; *r*=85.1				*p*<0.001; *r*=85.3				*p*<0.001; *r*=85.8 AIC=1157; RMSE=7.85; GCV=115.0			
	rain1lag2	0.01	0.00	0.00	0.01	0.00	0.01	0.00	0.00	0.00	0.00	0.00	0.00	0.00	0.06	0.00	0.00
Lag2	temp1lag2					0.17	0.00	0.11	0.22	0.15	0.00	0.10	0.21	0.16	0.00	0.11	0.21
	lagcase2									0.00	0.06	0.00	0.00	0.00	0.50	0.00	0.00
	interact2													0.00	0.01	0.00	0.00
	p and r correlation	*p*<0.001; *r*=74.0				*p*<0.001; *r*=75.2				*p*<0.001; *r*=75.8				*p*<0.001;*r*=76.4 AIC=1183; RMSE=1.29; GCV=171.0			
	rain1lag3	0.01	0.00	0.00	0.01	0.00	0.00	0.00	0.01	0.00	0.00	0.00	0.01	0.00	0.01	0.00	0.01
Lag3	temp1lag3					0.07	0.01	0.01	0.13	0.08	0.01	0.02	0.14	0.09	0.01	0.03	0.15
	lagcase3									0.00	0.11	0.00	0.00	0.00	0.01	0.00	0.00
	interact3													0.00	0.05	0.00	0.00

The full model also included an additional factor variable for month indicated that increasing monthly mean rainfall significantly preceded risks of increasing DF cases by 1–3 months, respectively (Table 3) while there was no significant association between temperature with DF cases except an inverse relationship by month 3. Numbers of DF cases of the two past months had significant autocorrelation with the number of DF cases of current month while numbers of DF cases at lag 3 had an inverse relationship with that of the current month. Interactions among rainfall, temperature, and DF cases at a lag time of 1–3 months always increase risk of increasing numbers of DF cases of the current month.

**Table 3:** Full multiple regression model coefficient and confidence intervals, Hanoi, 2002–2010

	*p*<0.001; *r*=92 AIC=1110; RMSE=33.63; GCV=45.2			
	
Model-5 Case	Coef.	P>z	95% CI	
_Iyear_2003	0.998	0.00	0.42	1.58
_Iyear_2004	1.013	0.00	0.45	1.57
_Iyear_2005	1.014	0.00	0.46	1.56
_Iyear_2006	2.176	0.00	1.62	2.74
_Iyear_2007	2.195	0.00	1.65	2.74
_Iyear_2008	1.323	0.00	0.77	1.87
_Iyear_2009	2.167	0.00	1.52	2.81
_Iyear_2010	2.277	0.00	1.68	2.87
temp1lag1	0.035	0.21	−0.02	0.09
rain1lag1	0.002	0.01	0.00	0.00
lagcase1	0.003	0.00	0.00	0.00
interact1	0.000	0.07	0.00	0.00
rain1lag2	0.003	0.00	0.00	0.01
temp1lag2	0.026	0.44	−0.04	0.09
lagcase2	0.001	0.03	0.00	0.00
interact2	0.000	0.00	0.00	0.00
rain1lag3	0.004	0.00	0.00	0.01
temp1lag3	−0.065	0.02	−0.12	−0.01
lagcase3	−0.001	0.04	0.00	0.00
interact3	0.000	0.00	0.00	0.00
_cons	−6.800	0.00	−8.42	−5.18

## Discussion

DF cases in Hanoi occurred annually and seasonally over the study period, 2003–2010, with recurrent peaks of DF cases in September and October. It also established a temporal relationship of recurrent patterns of rainfall and temperature preceding the outbreaks similar to Hii et al. (20, 11). In combination to autoregressive variables and incorporating lags in relationship up to 3 months, a correlation between observed and predicted dengue cases of above 90% was observed. This suggests a potential early warning system using these models with a lead time within this delay period (21).

Wilder-Smith and Schwartz from the Geo-Sentinel Surveillance Network had examined seasonality and annual trends for dengue cases among 522 returned travelers which indicated that dengue cases showed region-specific peaks for Southeast Asia (June, September), South Central Asia (October), South America (March), and the Caribbean (August, October) (22). DF has recently re-emerged globally with intensified epidemic and major epidemiological expansion since the 1980s, and has rapidly become a major epidemiological threat in Asia Pacific and South America (23). This current study also indicated that the number of DF cases increased significantly overtime. Hii et al. in their study of intensity and magnitude of dengue incidence in Singapore indicated that from 2000 to 2007, DF cases increased from 673 cases to the peak of 14,209 cases in 2005 (23). In Thailand, DF is on the rise; in 2012, Thailand recorded over 74,000 DF cases (24). Cambodia also observed an increasing trend of DF in which there were 15,597 cases between January and June in 2012 while that of 2011 was 4,604 cases, representing a 239% increase year-on-year (25). Worldwide there has been a 30-fold increase in cases of DF over the last 50 years (26).

Rainfall with stagnant water outdoors is considered a necessary condition for the breeding habitats of Aedes mosquitoes while temperature and humidity are, in combination, also a sufficient condition for effective development. Aedes mosquitoes adapt to harsh environmental conditions, which are sometimes produced by vector control programs or natural weather by laying their eggs in unusual outdoor habitats, or even on dry surfaces to wait up to several months for the appropriate amount of rainwater to hatch (16). Theoretical models of dengue transmission dynamics based on mosquito biology support the importance of temperature and precipitation in determining transmission patterns, but empirical evidence has been lacking. On a global scale, several studies have highlighted common climate characteristics of areas where transmission occurs (3). Meanwhile, longitudinal studies of empirical data have consistently shown that temperature and precipitation correlate with dengue transmission but have not demonstrated consistency with respect to their roles, and predictive performance with sufficient lead times. For example, cumulative monthly rainfall and mean temperature correlated positively with increased dengue transmission on the Andaman Sea side of Southern Thailand. On the Gulf of Thailand side, however, it was the number of rainy days (regardless of quantity) and minimum temperature that associated positively with incidence. Another study, farther north, in Sukhothai, Thailand, found that temperature had a negative effect on dengue transmission (27).

The current study indicated that there was a significant relationship between rainfall and DF cases. A graphic observation displayed that rainfall always peaks 2–3 months preceding the peaks of DF fever cases (Fig. 2) and model-5 showed that rainfall within 3 previous months at a level equal to or less than 450 mm was positively correlated with DF cases of the current month (Table 3). Our result is consistent with studies in Singapore and Brazil, which displayed that rainfall precedes risks of increasing DF cases by 1–5 months with higher risks being evident at 3–4 months (18, 19). Most recently, the outbreak of DF in Portugal which occurred during the unusually dry winter with rainfall predominantly in October through March then as of February 2013, resulted in over 2,000 cases among residents of Madeira in Portugal, most occurring between October and November 2012 (28). Hashizume et al. indicated that there was strong evidence for an increase in DF at high river levels during rainfall season. Hospitalizations increased by 6.9% for each 0.1 meter increase above a threshold (3.9 meters) for the average river level over lags of 0–5 weeks. Conversely, the number of hospitalizations increased by 29.6% (95% CI: 19.8, 40.2) for a 0.1 meter decrease below the same threshold of the average river level over lags of 0–19 weeks (29). This, once again, highlighted evidence of rainfall as a necessary condition for a DF outbreak explosion. Therefore, rainfall is still sensitively used as an indicator of a warning system for the DF outbreaks regarding stagnant water in natural puddles and canned food cover.

Assessment of risk of outbreak was mainly based on case, vector and virus surveillance which is already a part of the routine surveillance activity in many countries (30). The use of metrological data to predict and control dengue epidemics may not be a routine task for a health sector in many countries so far. Evidence of the relationship between rainfall and temperature from this study indicates that the integration of using climatic data into the existing surveillance activity may be beneficial to health workers working in the preventive medicine system in risk assessment and Information, Education and Communication (IEC) programs. In Hanoi, the IEC program to control DF outbreak was conducted in a way that if there was any outbreak of DF occurring in any district, then outbreak communication would be implemented to warn other districts following the IEC program delivered via loud speakers at health commune stations. Therefore, if using the integration approach, health workers should base levels of precipitations every month to make risk assessments along with looking at the number of cases and vectors measured from the surveillance system. Moreover, to prevent DF occurrence, the IEC program should be conducted in early April and last through October annually to remind people to destroy any stagnant puddles to eliminate breeding habitats of Aides mosquitoes after rainfall occurrence. This current study displayed that temperature precedes the risk of increasing DF cases by 1–2 months but this correlation was not statistically significant while an inverse correlation significantly happen at lag 3. The study conducted by Yan in Singapore also indicated that monthly mean temperature does not contribute to the prediction models of DF cases at any level (31).

However, temperature's role could be found to contribute to DF cases indirectly through interaction variables among rainfall, temperature, and DF cases which was significant at previous 2 months while there is a significantly inverse correlation between temperature and DF cases at lag 3. Studies in Singapore and Thailand showed that temperature precedes risks of increasing DF cases by 1–5 months and 6 months, respectively (23, 32).

In epidemiology, the infectious disease process chain of transmission always gives rise to autocorrelation. The autocorrelation arises as a natural feature of infectious disease systems as the number of new infections relates closely to the number of recent infections. A study by Joseph et al. in Puerto Rico (1988–1992) revealed a positive autocorrelation between the past and current DF (33). Hii et al.'s study in Singapore indicated that past DF cases from lag week 1 to 6 were considered to influence the occurrence of DF cases of the current week (34). Halide's study in city of Makassar indicated that the most important input variable in the prediction is the present number of DHF cases followed by the relative humidity 3–4 months previously (35). Autocorrelation also happened in the way of spatial autocorrelation in which geographical characteristics, density of population, and social factors contribute to the occurrence of DF cases in this area influence occurrence of DF cases in other areas especially in bordering areas. Suchithra indicated that there was a significant positive spatial autocorrelation of dengue incidence (36). This current study also indicated a partial correlation of numbers of DF cases significantly precede risks of increasing DF cases of the current month by 1–2 months. Moreover, this current study also showed that there were always DF outbreaks occurred in five bordering inner districts and two bordering outer districts every year where population density remains highest with lower social infrastructures. Mathuros in his study in Thailand implies that villages with geographical proximity shared a similar level of vulnerability to dengue (37).

Overall, the implication of the full model (model-5) is that whenever there was rainfall, DF cases with the appropriate temperature ranging from 15 to 30°C, these three factors would interact together preceding risks of an explosion of DF cases by 1–2 months. Therefore, any time when there are sporadic DF cases and rainfall occurs in warm weather, a risk of DF outbreak should be taken into account. However, more sophisticated automated early warnings systems could potentially give better predictive power.

Studies demonstrated that the extents of contribution of climate factors to the occurrence of DF cases varied remarkably. A study by Nazmul Karim indicated that the model incorporating climatic data of two-lag months explained 61% of variation in the number of reported dengue cases (38). As our study revealed a correlation of 92% (model 5), we could support that the discriminate power of these variables are substantial. Hii et al.'s study in Singapore indicated that climate factors contributed 84% to the occurrence of DF cases. However, Suwich Thammapalo in his study on climate factors and DF cases indicated that variability in incidence was explained mostly (14.7–75.3%) by trend and cyclic change and much less (0.2–3.6%) by independent climatic factors (39). This current study displayed that rainfall, temperature, autocorrelation of DF cases and their interaction contributed 92% to the correlation between predicted and observed DF cases (model-5, the fitness model; see Table 3).

However, limitations of the study are that there are many influential factors of DF epidemiology including urbanization, density of population, and globalization with increasing transport of goods not controlled for. Similarly, the predictive ability of the models does not capture impacts of co-circulation of different dengue virus types and other virus changes causing more disease relating to immunity processes. In general, factors of herd immunity, government vector control capacity, and changes in serotypes contribute to dengue epidemics. However, likewise climate changes, drought, and flood are conditional factors for increasing Aedes population size (40). There is a further need for studies on modeling contributing factors to DF including not only climatic factors but also social demographic and economic factors as well as a program of vector control to supply, and virus monitoring, to establish more accurate DF prediction in the future.

## Conclusion

DF in Hanoi occurred annually and seasonally in the period 2002–2010 in which a couple of DF cases appeared sporadically from December of the previous year to March of the next year, then increased gradually from April to July and afterward sharply peaked in September, October then decreased quickly in November and December. A new circle of DF cases would occur the year after. Monthly mean rainfall, temperature, DF cases, and their interaction from lag 1 to lag 3 contributed up to 92% correlation of predicted and observed DF cases in Hanoi. Monthly mean rainfall, autocorrelation, and interaction were statistical significantly related with monthly DF cases at lag 1–3 while temperatures were significantly related at lag 3.

## Policy recommendation

- To establish a more accurate and comprehensive model of DF prediction and early warning, additional research taking into account other forces or factors related to the urbanization, density of population, globalization with increasing transport of people and goods, herd immunity, government vector control capacity, and changes in serotypes beside climatic factors is needed. However, the findings suggest that the predictive power of weather factors and autocorrelation process are high already without this information, and that timely notifications to control DF outbreak and support immediate action in Hanoi by an information, communication, and education program focusing on training Hanoi residents may be achievable on this basis

## Figures and Tables

**Annex 2:**
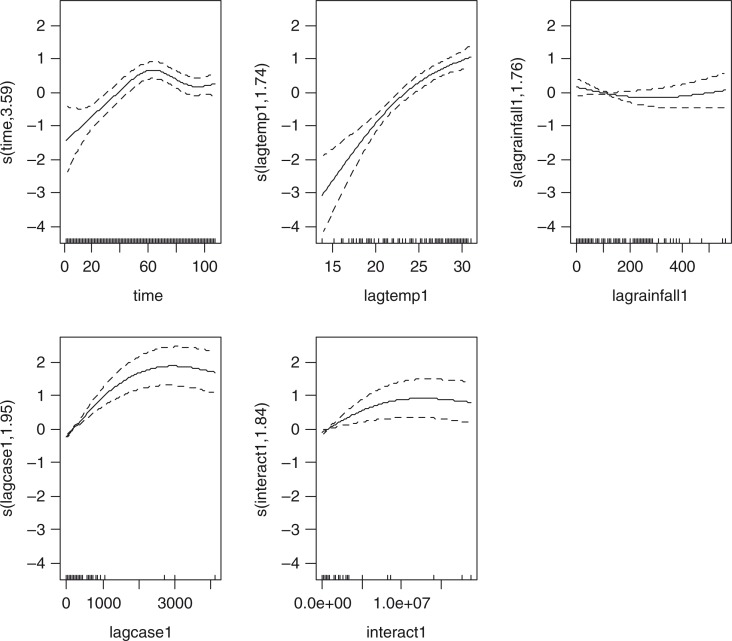
Graphs of model-4/lag1.

**Annex 3:**
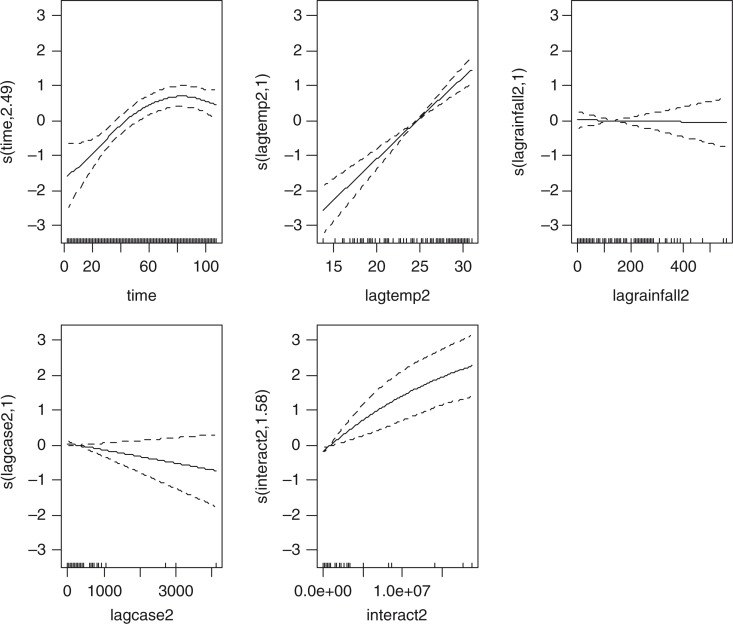
Graphs of model-4/lag2.

**Annex 4:**
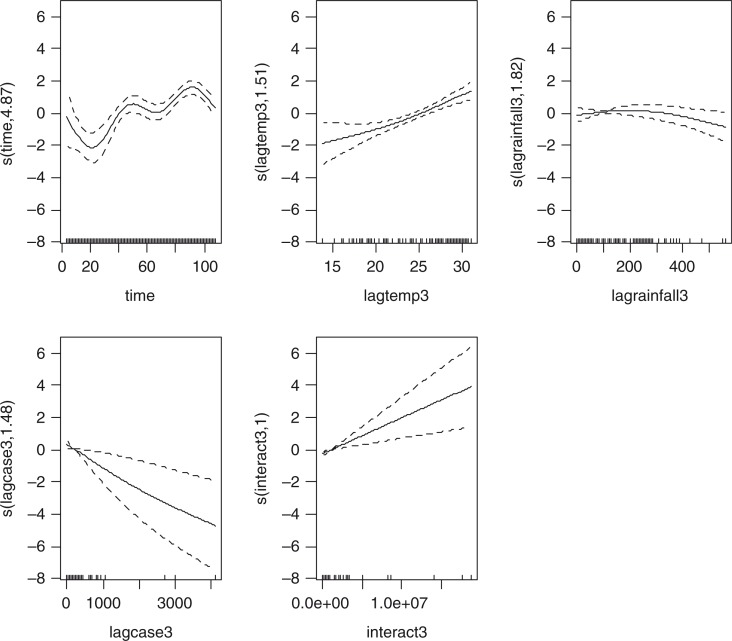
Graphs of model-4/lag3.

**Annex 5:**
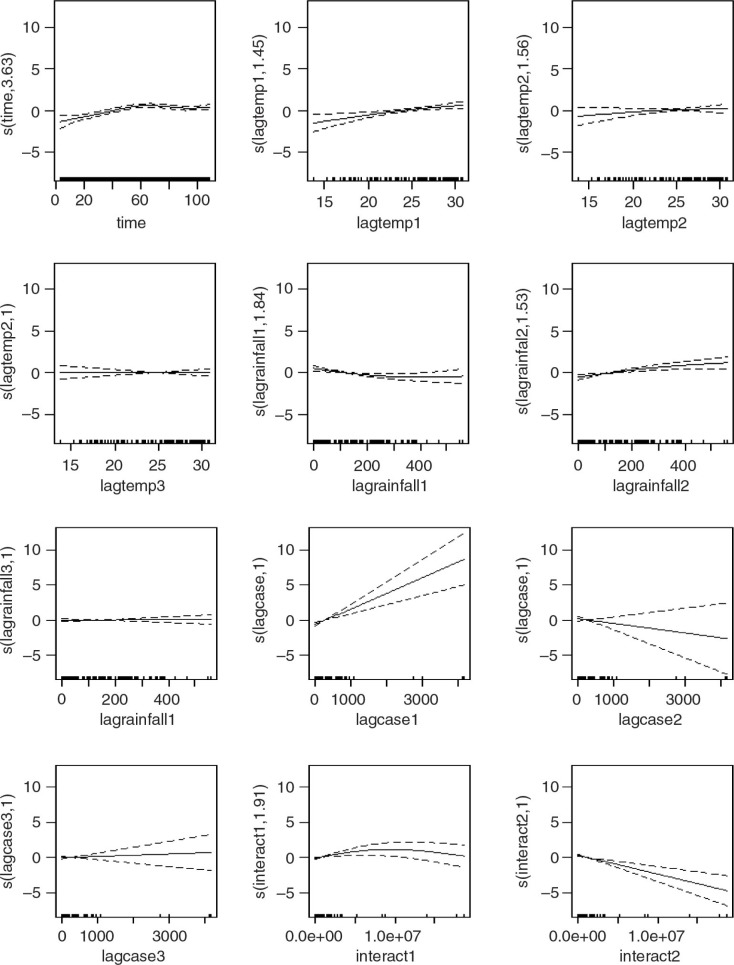
Graphs of model-5.

## Appendix

Annex 1: Definition of DF case

*Dengue fever:* Presence of high and continuous fever from 2 to 7 days and two or more of the following retro-orbital or ocular pain, headache, rash, myalgia, arthralgia, leukopenia, or hemorrhagic manifestations (e.g. positive tourniquet test, petechiae; purpura/ecchymosis; epistaxis; gum bleeding; blood in vomitus, urine, or stool; or vaginal bleeding) but not meeting the case definition of dengue hemorrhagic fever. Anorexia, nausea, abdominal pain, and persistent vomiting may also occur but are not case-defining criteria for DF.

*Dengue hemorrhagic fever* is characterized by all of the following:

- Fever lasting 2–7 days
- Evidence of hemorrhagic manifestation or a positive tourniquet test
- Thrombocytopenia (≤100,000 cells per mm^3^)
- Evidence of plasma leakage shown by hemoconcentration (an increase in hematocrit ≥20% above average for age or a decrease in hematocrit ≥20% of baseline following fluid replacement therapy), or pleural effusion, or ascites or hypoproteinemia.

*Dengue shock syndrome* has all of criteria for DHF plus circulatory failure as evidenced by:

- Rapid and weak pulse and narrow pulse pressure (>20 mm Hg), or
- Age-specific hypotension and cold, clammy skin and restlessness.

*Laboratory criteria for diagnosis for case definitions*

1. Confirmatory
  - Seroconversion from negative for dengue-specific serum IgM antibody in an acute phase (≤5 days after symptom onset) specimen to positive for dengue-specific serum IgM antibodies in a convalescent-phase specimen collected ≥5 days after symptom onset, *or*
  - Demonstration of a ≥4-fold rise in reciprocal IgG antibody titer or hemagglutination inhibition titer to dengue antigens in paired acute and convalescent serum samples, *or*
2. Criteria for Epidemiologic Linkage
  - Travel to an dengue endemic country or presence at location with ongoing outbreak within previous 2 weeks of dengue-like illness, *OR*
  - Association in time and place with a confirmed or probable dengue case.

DF cases collected in this study included all cases of DF, dengue hemorrhagic fever, and dengue shock syndrome who have laboratory confirmation or meet the criteria for epidemiologic linkage.

## Appendix

Annex 2: Sensitivity analysis using non-linear cubic functions instead of linear for model 4 and 5. Relationships are shown per model, lag and variable.
